# Case report: Radiofrequency-induced thermal burn injury in a dog after magnetic resonance imaging

**DOI:** 10.3389/fvets.2024.1364635

**Published:** 2024-05-13

**Authors:** Esther A. Lichtenauer, Koen M. Santifort, Niklas Bergknut, Iris van Soens, Martijn Beukers, Ines Carrera

**Affiliations:** ^1^IVC Evidensia Small Animal Referral Hospital Hart van Brabant, Neurology, Waalwijk, Netherlands; ^2^IVC Evidensia Small Animal Referral Hospital Arnhem, Neurology, Arnhem, Netherlands; ^3^Vet Oracle Teleradiology, Norfolk, United Kingdom

**Keywords:** skin folds, Shar-Pei, MRI complication, thermal injury, induction

## Abstract

A 10-year-old male Shar-Pei was referred for lethargy and proprioceptive deficits of the left thoracic limb. An magnetic resonance imaging (MRI) examination of the cervical spinal column and the brain was performed. The MRI examination of the brain was normal. A left-sided C3-C4 intervertebral disc extrusion with spinal cord compression was diagnosed. Medical treatment was elected. Within a week after the MRI examination, the dog presented with deep partial-thickness skin burn wounds in both axillae. Since the specific absorption rate had not exceeded the safety limits during any of the scans and no other procedures or circumstances were identified that could possibly have resulted in burn injuries, the thermal burn injuries were diagnosed as radiofrequency (RF) burns. The wounds healed by secondary intent over the next month. RF burns are the most reported complication in humans undergoing MRI but have not been reported in veterinary patients. Clinicians and technicians should consider the potential risk for RF burns in veterinary patients and take precautions regarding positioning of the patient and take notice of any signs of burn injury when performing follow-up examinations.

## Introduction

Magnetic resonance imaging (MRI) is deemed a relatively safe imaging modality. However, complications related to MRI examinations are reported more often in human patients than in veterinary patients. The most common complication, during MRI examinations, in human medicine is radiofrequency (RF) induced thermal burn injury.

Health agencies in the United Kingdom and the United States reported that RF burns account for ~50% of all MRI accidents (1, 2). These RF burns may be related to contact with conductive objects (e.g., specific clothing or ECG cables), skin-on-skin contact (e.g., skin folds or extremities in contact with the body) or contact with the bore (2). The reported severity of the burns varies from first degree (superficial-thickness) burns where only the epidermis is affected, to second degree (partial-thickness) burns where the epidermis and part of the dermis is affected, to third degree (full-thickness) burns where the epidermis and dermis are destroyed (2–5). By screening the patient prior to the MRI examination to identify conductive objects and by careful positioning of the patient during the MRI examination RF burns can be avoided (1).

Although technicians, neurologists and radiologists utilizing clinical MRI scans for veterinary patients may be aware of the complications reported in human medicine, there are, to our knowledge, no previous reports in veterinary medicine of MRI related RF burns in clinical cases. This case report documents the occurrence of RF burn injury in a Shar-Pei dog that underwent an MRI examination of the brain and cervical spinal cord.

## Case description

**Figure 1:**
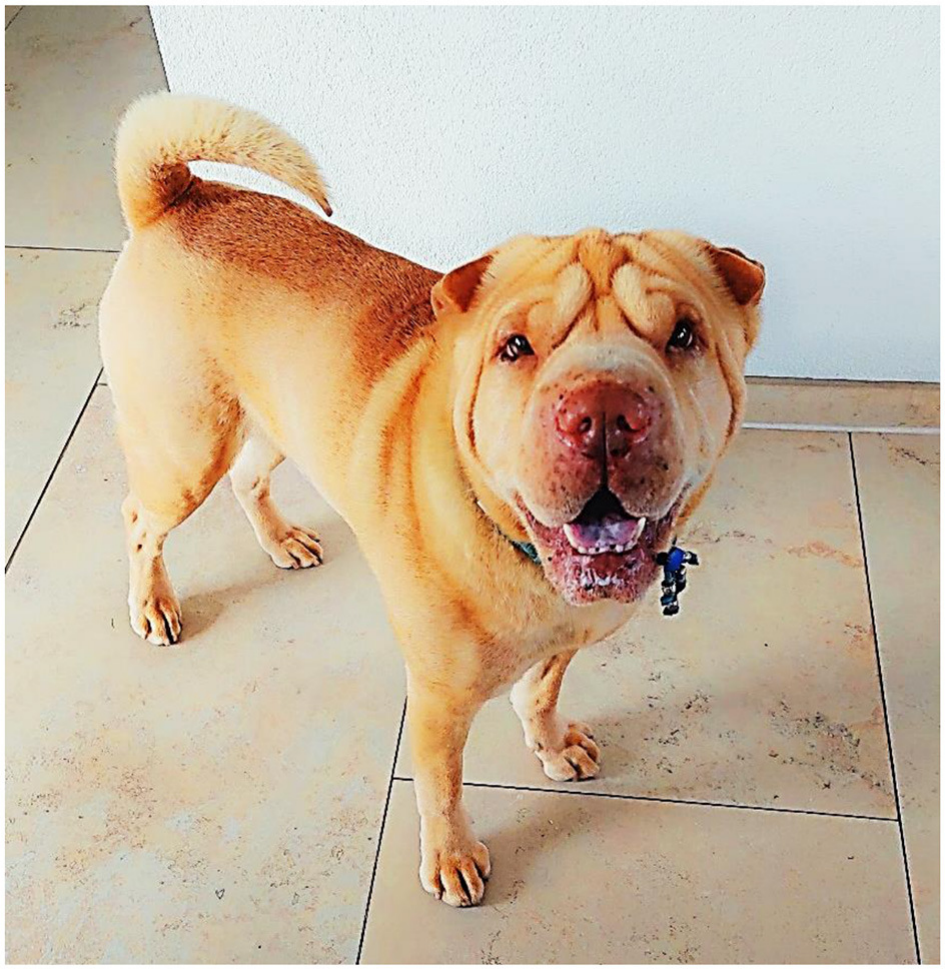
Overall appearance of the dog.

A 10-year-old male entire Shar-Pei dog was presented with one-day history of lethargy and stumbling on the left thoracic limb. General clinical examination was unremarkable, apart from the dog being more lethargic than expected. Also of note and particularly relevant to this report is the presence of thick skin with prominent skin folds, as is typical for this breed (6). Figure 1 shows the overall appearance of the dog. Neurological examination showed proprioceptive deficits in the left thoracic limb but with intact spinal reflexes. Palpation of the neck including passive movements did not show clear signs of hyperesthesia.

Based on the results of the neurological examination, a C1-5 myelopathy (left-sided) was suspected. Intracranial disease was not completely ruled out due to the ambiguous lethargy. Hence, an MRI examination of the brain and cervical spinal cord was planned. Pre-anesthetic blood work, including hematology, biochemistry, electrolytes, C-reactive protein (CRP) and pre-prandial bile acids did not reveal any clinically significant abnormalities.

Anesthesia of the dog for the MRI examination included premedication with 0.2 mg/kg butorphanol IV and 0.1 mg/kg midazolam IV and induction with 6 mg/kg propofol IV. Maintenance of the anesthesia was achieved with inhalant isoflurane. The patient was monitored during the anesthesia with capnography and an MRI compatible esophageal stethoscope.

**Figure 2:**
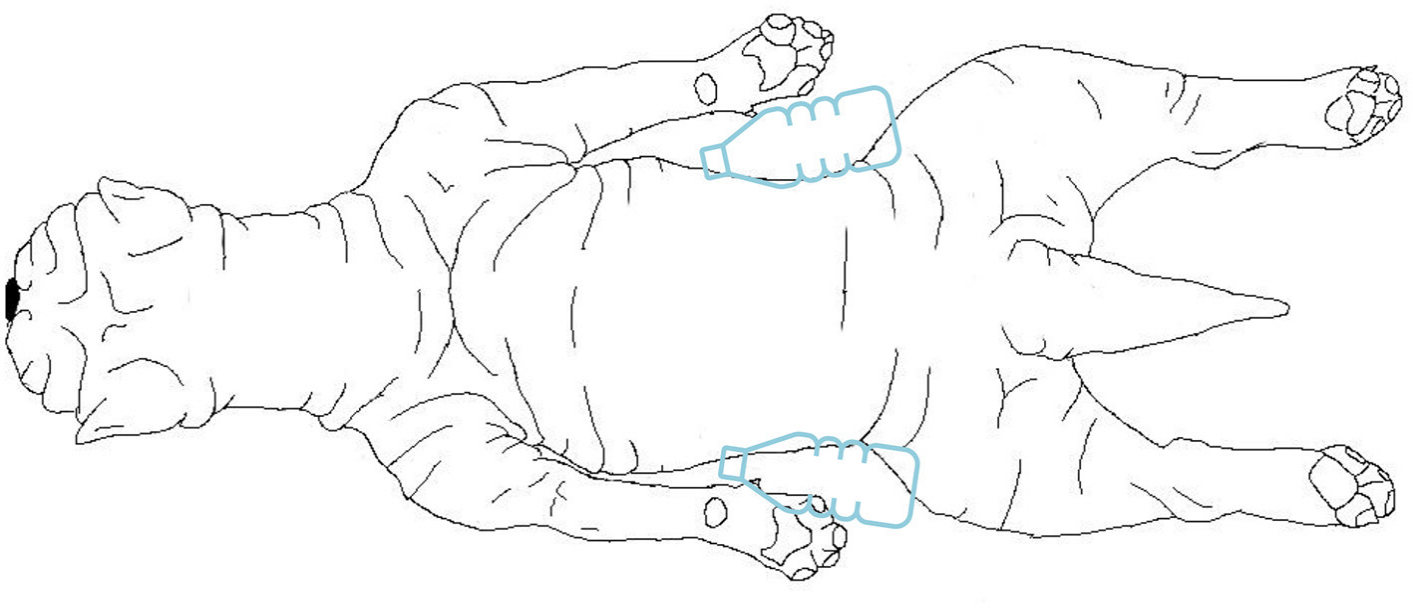
Drawing from a dorsal view of the positioning of the dog during the MRI examination. The dog was placed in sternal recumbency, with the thoracic limbs positioned backwards next to the trunk. Two bottles of warm water wrapped in a blanket were placed next to the patient's abdomen.

For the MRI scan (1.5T Canon Vantage Elan) the dog was placed in sternal recumbency, with the thoracic limbs positioned backwards next to the trunk (Figure 2). Two bottles of warm water wrapped in a blanket were placed next to the patient's abdomen and the patient was covered with a blanket. The following sequences were included for the study the brain: dorsal T1 weighted (W) inversion recovery (TR 2.4s, TE 18 ms, FOV 140 × 140 mm, slice thickness 3.0 mm, matrix 192 × 224, interslice gap 0.2 mm), sagittal T2W fast spin echo (FSE) (TR 5.5s, TE 120 ms, FOV 160 × 160 mm, slice thickness 3.0 mm, matrix 320 × 256, interslice gap 0.2 mm), axial T2W FSE (TR 5.9s, TE 90 ms, FOV 140 × 140 mm, slice thickness 3.0 mm, matrix 320 × 224, interslice gap 0.2 mm), T1W FSE (TR 0.4s, TE 10 ms, FOV 140 × 140 mm, slice thickness 3.0 mm, matrix 256 × 224, interslice gap 0.2 mm), susceptibility weighted imaging (TR 2,4s, TE 18 ms, FOV 200 × 200 mm, slice thickness 2.0 mm, matrix 320 × 304, interslice gap−1.0 mm), and diffusion-weighted imaging (TR 3,6s, TE 94 ms, FOV 200 × 225 mm, slice thickness 3.0 mm, matrix 160 × 256, interslice gap 1,5 mm) with apparent diffusion coefficient map. After intravenous contrast administration (gadolinium, 0.15 mmol/kg), axial T1W FSE (TR 0.4s, TE 10 ms, FOV 140 × 140 mm, slice thickness 3.0 mm, matrix 256 × 224, interslice gap 0.2 mm), T2W fluid attenuated inversion recovery (TR 8.0s, TE 120 ms, FOV 160 × 140 mm, slice thickness 3.0 mm, matrix 192 × 208, interslice gap 0.2 mm) and 3D T1W gradient echo (TR 10.4ms, TE 4.3 ms, FOV 180 × 160 mm, slice thickness 1.0 mm, matrix 180 × 160, interslice gap 0.0 mm) were acquired.

For the study of the cervical vertebral column the following sequences were included: dorsal short tau inversion recovery (TR 3.0s, TE 60 ms, FOV 200 × 300 mm, slice thickness 3.0 mm, matrix 192 × 288, interslice gap 0.3 mm), sagittal T2W FSE (TR 3495 ms, TE 110 ms, FOV 280 × 200 mm, slice thickness 2.5 mm, matrix 352 × 256, interslice gap 0.2 mm), T1W FSE (TR 663 ms, TE 10 ms, FOV 300 × 200 mm, slice thickness 2.5 mm, matrix 184 × 256 mm, interslice gap 0.2 mm), and axial T2W FSE (TR 5.6s, TE 115 ms, FOV 180 × 180 mm, slice thickness 2.5 mm, matrix 256 × 256, interslice gap 0.0 mm) and T1W FSE (TR 583 ms, TE 10 ms, FOV 180 × 180 mm, slice thickness 2.5 mm, matrix 192 × 192, interslice gap 0.0 mm) and 3D myelogram (TR 4s, TE 289 ms, FOV 220 × 220 mm, slice thickness 1.5 mm, matrix 224 × 272 mm, interslice gap 0.0 mm). Total scan time was ~75 min. During our study, the maximum specific absorption rate (SAR) was 0.39 W/kg during a sequence with a duration of <6 min. The average SAR per sequence was 0.12 W/kg and the duration of all sequences was maximum 6 min. The body temperature before and after the MRI scan was respectively 37.1°C and 38.0°C.

**Figure 3:**
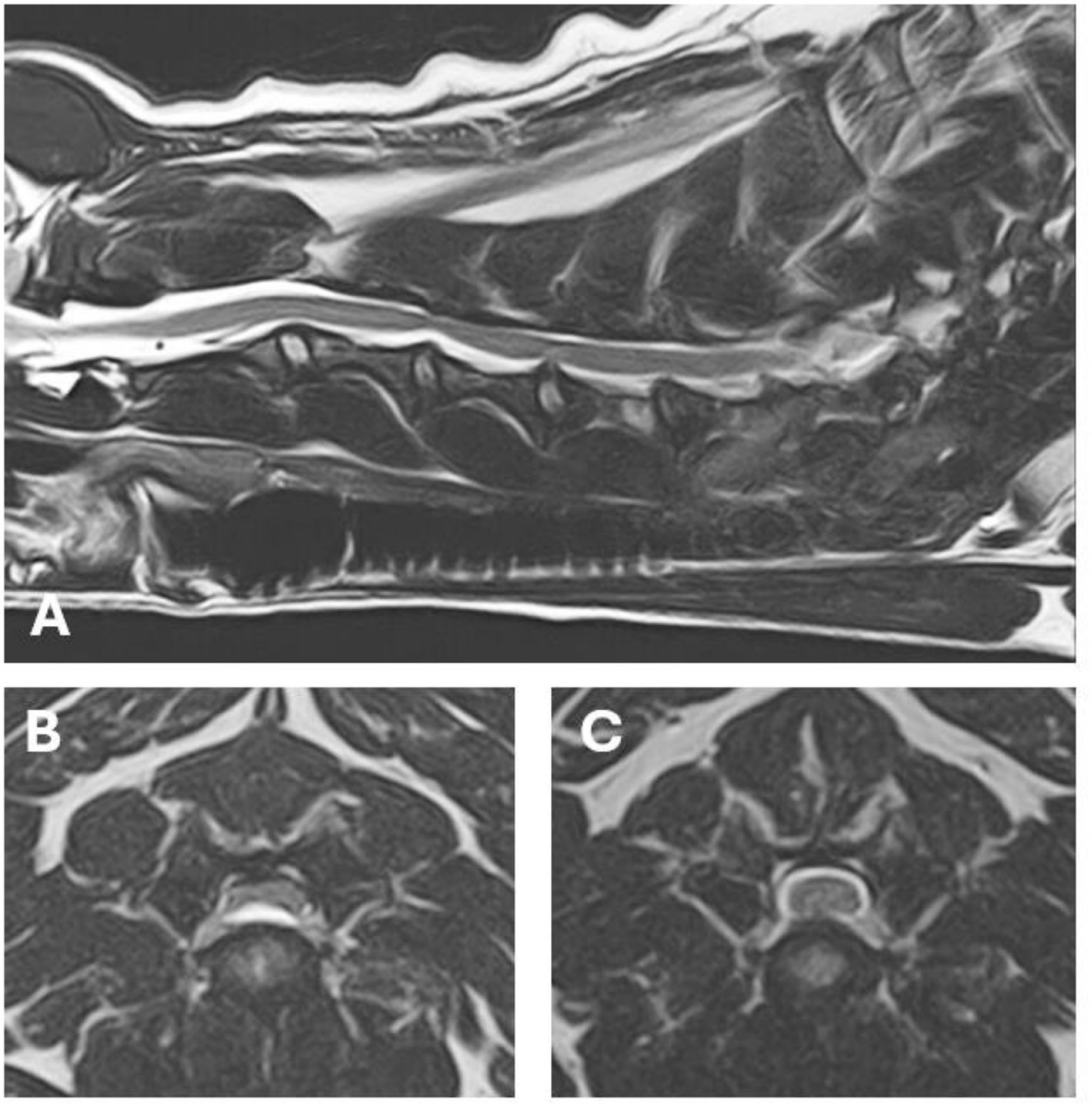
MRI images of the lesions found on MRI. **(A)** T2 weighted (W) sagittal image of the cervical spinal cord. **(B)** T2W axial image at the level of the C3–C4 hydrated nucleus pulposus extrusion with severe extradural spinal cord compression. **(C)** T2W axial image at the level of the minor C4–C5 intervertebral disc protrusion.

MRI showed severe extradural spinal cord compression at the level of the C3-C4 intervertebral disc, lateralized to the left side of the spinal canal (Figure 3). A hydrated nucleus pulposus extrusion was diagnosed. In addition, a minor protrusion of the C4-C5 intervertebral disc was found, without compression of the spinal cord. The MRI examination of the brain was unremarkable.

The owners elected conservative treatment and the patient was recovered from anesthesia. Meloxicam was prescribed at 0.1 mg/kg q24h (after a single starting oral dose of 0.2 mg/kg), together with gabapentin 10 mg/kg q8h and in addition to rest and controlled physiotherapy for 3 weeks.

**Figure 4:**
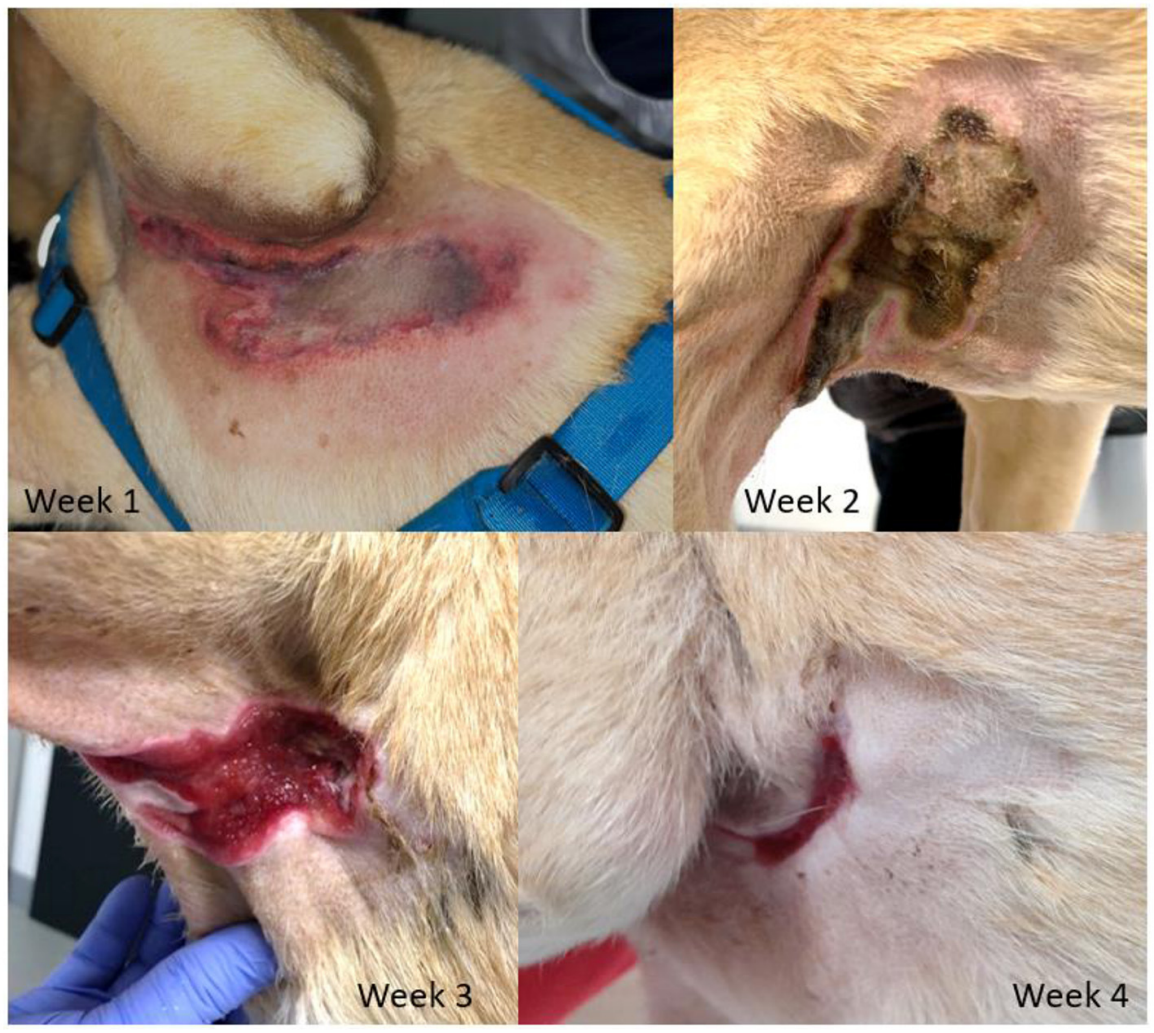
Photographical series of the burn wound in the left axillary region (sequence over a 4-week time span).

Several hours after the MRI the owner noticed a subcutaneous fluid filled pocket in the left axilla of the dog, which disappeared in the following days. Four days after the MRI the dog presented at the emergency service with a severe partial-thickness skin burn (Figure 4, week 1) and seven days after the MRI a second, but less severe, partial-thickness skin burn was noticed in the other axilla. The owner was questioned thoroughly and the procedures that had been performed were scrutinized. No other procedures or circumstances were identified that could possibly have resulted in burn injuries. Consequently, the thermal burn injuries were diagnosed as MRI related radiofrequency-induced burns.

After discussion with the owner about management options for the skin burns, conservative (non-surgical) treatment was elected. The burns were left to heal via secondary intention over the next few weeks, with regular revisits for bandaging and dressing with silver-sulfate (Figure 4). The owner still reported signs of pain despite the use of meloxicam and gabapentin. To improve the analgesia, tramadol was prescribed at 2 mg/kg q8h for 1 week in addition to the meloxicam and gabapentin. After 1 month both of the skin burns were almost healed and no further follow up was required. The meloxicam was discontinued and the gabapentin phased out over a course of 3 weeks. The amount of physical activity was slowly increased over a period of 1 month. At telephone follow-up 3 months after the initial consultation, no gait abnormalities, nor pain was reported by the owner, and the skin had almost completely healed.

## Discussion

There are four main types of burn injury: thermal injury, radiation injury, chemical injury, and electrical injury (7). MRI-related burn injuries fall in the first category, but consideration may be given to use a sub-classification due to its specific nature, such as “radiofrequency-induced thermal burn injury.” In our case, the owner was questioned to identify any other plausible causes of the burn injuries, including: circumstances at home; any procedures that might have been performed between the MRI and the presentation for the burn injuries; possible exposure to chemicals; contact with radiators; exposure to radiation or electrical appliances of any kind. None were identified. In any case, the sites where the burns occurred are not likely to have been exposed to other likely causes for burn injuries (such as contact with radiators). Finally, a causal relationship between the MRI and the burn injuries is supported by the time relationship between the MRI study and presentation for the burn injuries. All in all, we concluded that the burn injuries in our patient were MRI related radiofrequency-induced burns. To the authors' knowledge, this has not been documented before in veterinary medicine and is not reported in reviews on the subject (7, 8).

The thermal burn injury in the dog reported here may be classified as a local, partial-thickness burn injury. This type of burn injury may take 24–48 hours to become apparent for owners or veterinarians. Partial-thickness skin burns can heal within 1–3 weeks by re-epithelialization from hair follicles and sebaceous glands. A few days after the burn injury an eschar may be formed. Partial-thickness skin burns heal with minimal scar formation. Burn injuries can have serious local and systemic consequences for a patient and need to be addressed with care. Fortunately, the reported patient here did not experience any further significant complications during treatment of the wound (7–9).

MRI related thermal burn injuries are the most common reported adverse event in humans after an MRI scan (2) and numerous human cases have been reported in the literature (3–5, 10–15). Causes for thermal burn injuries during an MRI scan can be skin-to-skin contact, bore contact, contact with an object, not RF related or unclear (2).

Thermal burn injuries caused by radiofrequency pulses can occur when there is contact between skin and a conductive object or direct skin-to-skin contact. There are several proposed mechanisms for the occurrence of RF burns, such as inductive heating, heating of a resonant loop and the “antenna effect.” With inductive heating the RF electromagnetic field causes currents to flow through a conductive object which will heat the object. This phenomenon is called ohmic heating. When these currents flow in a loop that is in a resonant condition, a resonant loop forms and the heating will become significant. With the “antenna effect” an elongated conductive object forms a resonant loop (13, 15, 16). Dempsey et al. have shown that the temperature of a conductive object can rise by 0.6°C with inductive heating, and by 61.1°C and 63.5°C with heating of a resonant loop or a resonant antenna, respectively (16).

RF heating increases with a stronger magnetic field. Theoretically, RF heating is proportional to the square of the static magnetic field (17). Now that the magnetic field strength of MRI devices are generally increasing, and 1.5T or 3.0T are becoming more commonly used in veterinary practice, there is a potentially increasing risk for RF burns.

Theoretical heating of the tissues of the patient caused by the RF pulses is evaluated by the SAR, which is depicted in watts per kilogram bodyweight. The SAR can be calculated taking into account the patients height, weight, and the scan settings such as for example RF pulse frequency and the angulation of the RF magnetic field (18). There are guidelines with upper allowable SAR values for safe MRI examinations in humans. The maximal SAR that is allowed is <4.0 W/kg over 6 min. With this SAR, the body temperature of the person will rise by 1°C maximum (1). For animals, no safety limits have been established for the SAR. During our study, the maximum SAR was 0.39 W/kg during a sequence with a duration of <6 min. The average SAR per sequence was 0.12 W/kg and the duration of all sequences was maximum 6 min. This could not have significantly contributed to the thermal burn injuries. However, when a resonant conductive loop was formed, focal overheating could have occurred in our patient, even when the SAR did not exceed the upper limit.

As is indicated with the SAR, the time of an MRI examination itself may be one of the factors involved in predicting the risk for thermal burn injuries in patients. In this case, the brain as well as the cervical spinal cord was scanned. Reducing scan time (e.g., by leaving out certain sequences that may not be necessary in every case), reduces the total energy ‘transmitted' to the body of the patient. It has been shown that ‘rapid' protocols for brain imaging yield comparable differential diagnoses compared to a “full” brain protocol (19). Both the occurrence and severity of burn injuries may be influenced by total scanning time.

In our case, the patient was of a dog breed known for its loose and thick skin with prominent skin folds (6). Also, the patient had thin fur in its axillae. The skin and fur characteristics, together with the positioning of the patient during the MRI examination make it likely that skin-to-skin contact occurred between skin folds in the axillae. There was no contact between the dog and conductive objects or the bore. Even though the patient underwent an extensive MRI examination, the SAR had not been exceeded. Based on these data, we concluded that the thermal burn injuries reported in our patient must be RF thermal burn injuries by skin-to-skin contact. It is also possible that the thick skin of the Shar-Pei and its histological and molecular characteristics predispose this breed to RF thermal burn injury occurrence. Although this is merely a theory, we would propose to carefully monitor dogs of this breed undergoing MRI studies for RF thermal burn injuries and take precautionary measures as discussed below.

The fact that our patient was under general anesthesia and thus not able to report any pain may have contributed to the occurrence of the RF burns. Human patients that are conscious during the MRI examination can press the alarm-button when they feel pain or a hot sensation. This safety feature is unfortunately not possible in veterinary patients. During the monitoring of the anesthesia of our patient, a temporary increase in the heartrate was noticed. In retrospect we assume that this may have been caused by pain due to the occurrence of the RF burns.

Precautions must be taken to prevent RF burns. In human medicine MRI safety guidelines are available (1, 20). In these guidelines recommendations are made on how to prevent RF burns. No specific protocols exist for the veterinary patient. By extrapolation from the human safety guidelines we propose some considerations listed in Table 1 to prevent thermal burn injuries when performing MRI studies in veterinary patients.

**Table 1:** Proposed considerations to reduce the risk of MRI-induced radiofrequency burns in veterinary patients.

Screen patients	Screen patients for implants, devices and other metallic objects. Unknown objects should be assumed unsafe.
Screen objects	Screen objects in the scan room for MRI safety.
No wearables	Remove all leashes, collars, clothing, anti-parasitic collars, etcetera, from the patient
Positioning	Position the patient to avoid skin-to-skin contact
Padding	Place protective padding in between areas where there is a risk for skin-to-skin contact, contact with the bore, coils or cables.
Cables	Route cables out of the scanner in a straight line and don't let cables touch the patient
SAR	Follow the safety limits of the SAR and use the lowest SAR as possible.
Monitor patient	Monitor the patient; e.g., increases in respiratory rate or heart rate may indicate a painful sensation caused by a RF burn.

In conclusion, MRI related radiofrequency-induced thermal burn injuries can occur in dogs undergoing standard MRI examinations using a 1.5T scanner. Clinicians and technicians should be aware of the potential risk of this complication and 1/ take precautions to prevent its occurrence 2/ carefully inspect (at-risk) patients after MRI studies to take adequate measures in case of burn injuries.

## Data availability statement

The raw data supporting the conclusions of this article will be made available by the authors, without undue reservation.

## Ethics statement

Ethical approval was not required for the studies involving animals in accordance with the local legislation and institutional requirements because the data used in this case report was obtained during a medical consultation requested by the owner of the animal. Written informed consent was obtained from the owners for the participation of their animals in this study.

## Author contributions

EL: Conceptualization, Funding acquisition, Investigation, Project administration, Visualization, Writing – original draft, Writing – review & editing. KS: Conceptualization, Supervision, Visualization, Writing – original draft, Writing – review & editing. NB: Conceptualization, Investigation, Supervision, Writing – review & editing. IS: Supervision, Writing – review & editing. MB: Visualization, Writing – review & editing. IC: Supervision, Visualization, Writing – review & editing.
